# Supplementary material for: Transcriptome Analysis Reveals Vimentin-Induced Disruption of Cell–Cell Associations Augments Breast Cancer Cell Migration
Source: Cells. 2022 Dec 13;11(24):4035. doi: 10.3390/cells11244035 (PMC9776984; doi:10.3390/cells11244035)
Supplement: Supplementary file 1 [file cells-11-04035-s001.zip › DEGs and enrichment analysis.zip!/DEGs and enrichment analysis/WT_VIMvsVector KEGG/DOWN/WT_VIMvsVector_DOWN.pathway_map.zip!/WT_VIMvsVector_DOWN.pathway_map/src/hsa04360.html]

hsa04360


- hsa:59277

- Down regulated genes

ENSG00000074527(-2.47423263734555)

- hsa:9037

- Down regulated genes

ENSG00000112902(-2.34836214109902)

- hsa:9901

- Down regulated genes

ENSG00000196220(-2.23876692290155)

- hsa:27289

- Down regulated genes

ENSG00000172602(-2.20834475156536)

- hsa:27289

- Down regulated genes

ENSG00000172602(-2.20834475156536)

Close
